# Detailed Characterization of the Conversion of Hardwood and Softwood Lignin by a Brown-Rot Basidiomycete

**DOI:** 10.1021/acs.biomac.4c01403

**Published:** 2025-01-06

**Authors:** Morten Rese, Gijs van Erven, Romy J. Veersma, Gry Alfredsen, Vincent G. H. Eijsink, Mirjam A. Kabel, Tina R. Tuveng

**Affiliations:** 1 Faculty of Chemistry, Biotechnology, and Food Science, Norwegian University of Life Sciences (NMBU), P.O. Box 5003, Ås 1433, Norway; 2 Wageningen Food and Biobased Research, Bornse Weilanden 9, Wageningen 6708 WG, The Netherlands; 3 Laboratory of Food Chemistry, Wageningen University & Research, Bornse Weilanden 9, Wageningen 6708 WG, The Netherlands; 4 Department of Wood Technology, Norwegian Institute of Bioeconomy Research, P.O. Box 115, Ås NO-1431, Norway

## Abstract

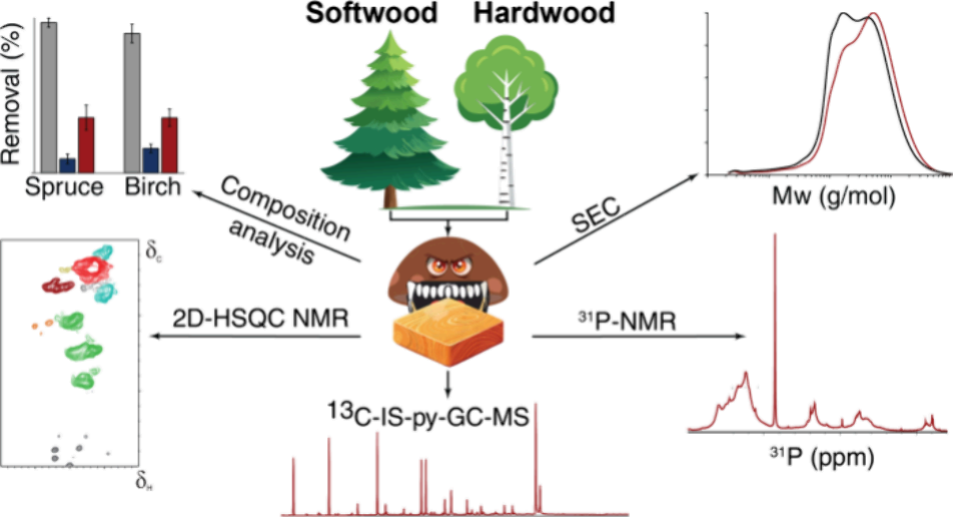

Wood-degrading brown-rot fungi primarily target carbohydrates, leaving the lignin modified and potentially valuable for valorization. Here, we report a comprehensive comparison of how *Gloeophyllum trabeumin vitro* degrades hardwood and softwood, which have fundamentally different lignin structures. By harnessing the latest advancements in analytical methodologies, we show that *G. trabeum* removes more lignin from wood (up to 36%) than previously reported. The brown-rot decayed lignin appeared substantially C_α_-oxidized, *O*-demethylated, with a reduction in interunit linkages, leading to formation of substructures indicative of C_α_-C_β_, β-*O*, and *O*-4 cleavage. Our work shows that the *G. trabeum* conversion of hardwood and softwood lignin results in similar modifications, despite the structural differences. Furthermore, lignin modification by *G. trabeum* enhances the antioxidant capacity of the lignin and generates an extractable lower molecular weight fraction. These findings improve our understanding of lignin conversion by brown-rot fungi and highlight their biotechnological potential for the development of lignin-based products.

## Introduction

In nature microorganisms play a pivotal role in the carbon cycle, working symbiotically to degrade lignocellulosic biomass.^1,2^ A better understanding of microbial wood degrading systems provides valuable knowledge on environmental carbon dynamics and may help unleashing the potential of using biomass components as a sustainable resource for producing value-added chemicals and products.

Lignocellulose is primarily composed of cellulose, hemicellulose, and the heterogeneous aromatic polymer lignin. Independent of the plant species, cellulose is present as a homopolymer of glucosyl units linked via β-(1 → 4) glycosidic bonds. In contrast to cellulose, the hemicellulose and lignin structures are fundamentally different across plant taxa. Softwood hemicellulose is primarily composed of a glucomannan backbone, substituted with galactosyl units, and arabinoglucuronoxylan, a xylan backbone substituted by arabinosyl and 4-*O*-methylglucuronyl units. Hardwood hemicellulose consists primarily of a xylan backbone, substituted with 4-*O*-methylglucuronyl units.^3−5^

Representing up to 30% of the dry matter in terrestrial plants, lignin is a major component of lignocellulosic biomass and the most abundant reservoir of aromatic carbon on Earth.^1,6^ During its biosynthesis, lignin is built by radical coupling of the phenylpropanoids *p-*coumaryl, coniferyl and sinapyl alcohol, giving rise to *p-*hydroxyphenyl (H), guaiacyl (G) and syringyl (S) subunits, respectively. The H, G, and S subunits are coupled through various aryl-ether and carbon–carbon linkages, with the β-*O*-4 aryl ether motif as the most abundant interunit linkage.^7^ Within the domain of vascular plants, softwood lignins are primarily composed of G units, while hardwood lignins typically consist of more S than G units. The two main lignin types also differ in terms of the relative abundance of interunit linkages. Softwood lignins are typically enriched in β-5 and other C–C condensed linkages because of their guaiacyl nature. To conquer the natural recalcitrance of lignin, microorganisms, primarily fungal species, have developed intricate enzymatic and nonenzymatic strategies to modify and/or degrade lignin.^8^ Although significant progress has been made in understanding fungal lignin degradation, many aspects of these complex processes remain poorly understood. Recent advancements in lignin analytical techniques now enable a more detailed exploration of lignin degradation.

Basidiomycete fungi are recognized for their ability to convert lignocellulose, and species within this fungal division rely on vastly different conversion strategies and mechanisms. Based on this variation, basidiomycete species are commonly classified into brown-rot or white-rot fungi.^9^ Selective white-rot fungi cause wood decay that is characterized by a lighter-colored residue, resulting from selective delignification that leads to a cellulose-enriched material.^10^ This selective process is catalyzed by high-redox potential heme-peroxidases and laccases.^11^ In contrast, brown-rot fungi selectively degrade polysaccharides, leaving a brown residue rich in oxidized lignin.^12−14^ The current model used to explain lignin oxidation by brown-rot fungi entails the generation of extracellular hydroxyl radicals (·OH) through the nonenzymatic Fenton reaction. These radicals diffuse into the lignocellulosic matrix causing nonselective oxidation of polysaccharides and lignin.^15−17^ Given the traditional focus on valorization of cellulose and other polysaccharides in biorefineries, white-rot fungi have sparked particular biotechnological interest, due to the ability of certain species to selectively remove lignin from biomass.^18,19^ With the rising interest in lignin-focused valorization concepts, brown-rot fungi may be valuable in the biotechnological context as well since their conversion of lignocellulose results in a lignin-enriched residue amenable to further processing. This calls for a better understanding of brown-rot, especially related to the selectivity of polysaccharide and lignin conversion and the detailed structural features of the residual lignin.

Although brown-rot fungi typically cause less extensive lignin structural alteration as compared to white-rot fungi, multiple studies have demonstrated significant lignin modifications.^20−22^ The described modifications include *O*-demethylation of aromatic structures and cleavage of interunit linkages.^23−25^ However, different brown-rot fungal species and wood types have been used in these studies. Therefore, it remains unclear how variations in wood types with inherently distinct lignin structures affect the pattern of lignin modifications caused by brown-rot fungi. Previous studies also indicate that brown-rot fungi remove only a small portion (up to 16%) of the lignin.^20−22^ These findings are based solely on lignin quantification using traditional gravimetric methods, which have known limitations.^26−28^ Therefore, it is essential to revisit the issue of lignin modification by brown rots using the latest analytical lignin analysis methods to determine the extent of lignin modification and removal. Additionally, the potential added value of new functionalities in the structure of the modified lignin for valorization purposes should be explored.

Recent advancements, particularly the combined application of pyrolysis gas chromatography–mass spectrometry with uniformly ^13^C-labeled lignin applied as internal standard (^13^C-IS-py-GC-MS) and two-dimensional heteronuclear single quantum coherence nuclear magnetic resonance (2D-HSQC NMR) spectroscopy, has shed new light on the complex structure of lignin and the mechanisms employed by white-rot fungi for its degradation.^29−31^ In this study, we leverage this combined approach (^13^C-IS-py-GC-MS and 2D-HSQC NMR) to gain a comprehensive understanding of the extent of lignin removal and structural modification, by following and comparing the conversion of hardwood and softwood lignin by the well-established model brown-rot fungus *Gloeophyllum trabeum*. Finally, we investigate how *G. trabeum* can generate a more reactive lignin fraction, enhancing its suitability for valorization.

## Materials and Methods

### Brown-Rot Decay of Wood

Wood blocks with the dimension of 5 × 10 × 30 mm were cut from one board of Norway spruce (*Picea abies* (L.) H. Karst., softwood) or Downy birch (*Betula pubescens* Ehrh., hardwood), followed by autoclaving at 121 °C for 20 min. The wood blocks were inoculated using a liquid culture of the brown-rot fungus *Gloeophyllum trabeum* (Pers.) Murrill, strain CTB 863A, a commonly used model fungus.^32,33^ Following growth on 4% (wt/vol) Difco malt agar medium (VWR, Oslo, Norway) for 7 days at 22 °C and 70% relative humidity, plugs with a diameter of 4 mm containing actively growing mycelium were transferred to a sterile malt solution containing 4% (wt/vol) Difco malt (VWR). After 2 weeks of incubation the liquid culture was homogenized with a tissue homogenizer (Ultra-Turrax T25; IKA Werke GmbH & Co. KG, Staufen, Germany).

To produce brown-rot decayed wood, a modified E10–22 soil/block test was used.^34^ Prior to inoculation, the wood blocks were placed on Petri dishes (TC dish 100, standard; Sarstedt AG Göttingen, Germany) containing 2/3 compost soil and 1/3 sandy soil and were adjusted to 95% of the soils water-holding capacity according to the procedure given in ENV 807.^35^ The soil was sterilized in an autoclave for 3 × 60 min at 121 °C and 20 g sterile soil was added to each Petri dish under sterile conditions. A sterile plastic mesh was used to avoid direct contact (i.e., preventing water logging) between the wood blocks and the sterile soil (Figure S1). Each wood block was inoculated by adding 1 mL of homogenized liquid culture.

The Petri dishes, each containing four wood blocks were incubated at 22 °C and 70% relative humidity for 18 weeks. Sterile water was added under sterile conditions to regain initial soil moisture content. For a set of spare samples initial dry weight before decay was recorded (dried at 103 °C for 20 h) and the inoculated wood blocks were harvested at three-week intervals starting at week 12. Mass loss quantification was included in this study to ensure that the wood was harvested at the end stage of conversion (i.e., mass loss around 70%). Surface mycelia were removed with paper wipes, and the wood was dried at 103 °C for 18 h to measure final dry weight. Mass loss was calculated according to the following equation:
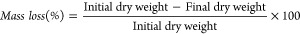


All samples were harvested after 18 weeks. The fungal mycelia were removed with paper wipes and the wood was dried for 18 h at 40 °C followed by grinding using an IKA-mill (Staufen, Germany) with a 1 mm mesh, resulting in a fine powder. These samples are hereafter called brown-rot decayed wood.

### Sound Wood Samples

Untreated spruce and birch was dried at 40 °C for 24 h before milling using a SM300 mill (Retsch, Haan, Germany) operated at 1500 rpm with a 2 mm screen. The milled samples were sieved (0.2 mm) and wood particles passing through the sieve (i.e., < 0.2 mm in size) were used in subsequent analysis (hereafter referred to as sound wood).

### Compositional Analysis

Sound wood samples of spruce and birch were subjected to consecutive extractions with ethanol and water followed by drying at 60 °C for 24 h. The content of structural carbohydrates, acid soluble lignin (ASL), acid insoluble lignin (AIL), and ash in brown-rot decayed wood and ethanol extracted sound wood were determined according to the National Renewable Energy Laboratory standardized protocol (NREL/TP-510–42 618) described by Sluiter et al.^36^ The total lignin content (ASL+AIL) is referred to as Klason lignin. All samples were analyzed in triplicate. For the sugar compositional analysis, samples were incubated with 72% H_2_SO_4_ at 30 °C for 1 h, followed by 4% H_2_SO_4_ at 121 °C for 1 h. Sugar recovery standards (d-galactose, d-glucose, d-mannose, d-xylose, and d-arabinose, 1.5 g L^–1^ of each) were treated with H_2_SO_4_ at a final concentration of 6% and incubated at 121 °C for 1 h.

Quantification of monomeric sugars was performed using high-performance anion-exchange chromatography with pulsed amperometric detection on a Dionex ICS 6000 system (Thermo Fisher Scientific, Waltham, MA, USA). The system was equipped with a CarboPac PA210 analytical column (2 × 150 mm) and a CarboPac210 guard column (2 × 30 mm). With a flow rate set to 0.2 mL min^–1^ and 1 mM KOH as eluent, products were eluted isocratically over 15 min, with pulsed amperometric detection (PAD). Chromatograms were analyzed using the Chromeleon 7.2.9 software (Thermo Fisher Scientific, Waltman, MA, USA).

ASL was determined with a Cary 60 UV–vis spectrophotometer (Agilent Technologies, Santa Clara, CA, USA) at 205 nm, using an extinction coefficient of 110 g L^–1^ cm^–1^.^37^ The nitrogen content (% N) in brown-rot decayed wood and in the AIL fraction of brown-rot decayed wood was determined in duplicate using the DUMAS combustion method on a Vario El Cube elementanalysator (Elementar Analysensysteme GmbH, Hanau, Germany). For calculation of the protein content a conversion factor of 6.25 was used.^38^ Since we only detect nitrogen in the brown-rot decayed wood we assume that this nitrogen originates from residual fungal biomass. All nitrogen is assumed to originate from protein although we cannot rule out that other nitrogen containing compounds contribute to the nitrogen content. Of note, % N in sound wood was below the limit of detection (0.05%). Since protein can lead to errors in the determination of AIL, the calculations of AIL were corrected for protein content.

Calculation of carbohydrate and lignin removal was done according to the following equation:
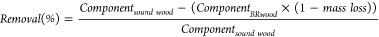
with “component” referring to the amount (grams) of carbohydrates or lignin in either sound wood or brown-rot decayed wood (BR wood). The equation calculates the percentage of the component removed during treatment by comparing the original amount in the sound wood to the amount remaining in the brown-rot decayed wood, adjusted for the overall mass loss (expressed as grams of decayed wood per gram of sound wood). Removal is expressed as a percentage of the component’s original weight in the sound wood.

### Quantitative ^13^C-IS-Pyrolysis-GC-MS

Pyrolysis coupled to gas chromatography with high resolution mass spectrometry detection (py-GC-MS) (Exactive Orbitrap, Thermo Scientific, Waltham, MA) was performed, as described previously,^39^ using a on a Trace 1300 GC equipped with an Agilent VF-1701 ms fused-silica column (30 m × 0.25 i.d., 0.25 μm film thickness; Thermo Fisher Scientific Inc., Waltham, MA, USA) for chromatographic separation. A split ratio of 1:133 was applied for the first 5 min, followed by a reduced split ratio of 1:13.3 for the remaining 52 min. The reduction minimized helium consumption while maintaining method performance. For spruce and birch samples (both sound and brown-rot decayed wood), ^13^C-douglas fir lignin or ^13^C-willow lignin isolates, obtained from uniformly ^13^C labeled douglas fir (97 atom % ^13^C) and uniformly ^13^C labeled willow (96 atom % ^13^C) (IsoLife, Wageningen, The Netherlands), were used as internal standards, respectively.^39^ Hereto, 10 μL of 1 g L^–1 13^C-lignin, dissolved in a 50:50 mixture of chloroform and ethanol (v/v), was added as an internal standard to 80 μg carefully weighed samples (XP6 excellence-plus microbalance; Mettler-Toledo International Inc., Columbus, OH, USA). Samples were analyzed in triplicate. Lignin-derived pyrolysis products were analyzed using full mass spectrometry (MS) mode. For each compound, the monitoring was focused on the most abundant fragment, whether the compound was nonlabeled or uniformly labeled with ^13^C (Table S1). Pyrograms were processed using TraceFinder 4.0 software (Thermo Fisher Scientific). Lignin contents and relative abundances of lignin-derived pyrolysis products were calculated as described previously.^39^ Catechol and methoxycatechol pyrolysis products in brown-rot decayed wood were (tentatively) identified based on retention time, exact mass, fragmentation, and available standards (Table S2). Relative response factors for catechol and methoxycatechol pyrolysis products were estimated using the structurally closest analogs.

### Isolation of Lignin for Detailed Structural Characterization

To characterize the lignin structure in the sound wood and brown-rot decayed wood, lignin isolates were prepared by enzyme treatment as described previously.^40^ In brief, dry milled wood samples were further planetary ball milled using settings as published.^30^ Finely milled samples (800 mg) were subsequently dispersed in 20 mL of 50 mM sodium acetate, pH 5.0, and treated with commercial preparations of cellulase (Cellylysin, Sigma-Aldrich, St Louis, MO, USA), 25 mg/g substrate dose), xylanase (Viscostar 150 L, Dyadic Jupiter, FL, USA, 150 μL/g substrate dose) and for softwood samples additionally with mannanase (Gamanase 1.5 L [Novozymes, Bagsværd, Denmark], 50 μL/g substrate dose), for 72 h at 40 °C to degrade the polysaccharides present. Residues after the enzymatic treatment were acidified with 2 M HCl, followed by centrifugation (4700x*g*, 5 min, 20 °C), and washed twice with 10 mL of Milli-Q water acidified to pH 2.0 with hydrochloric acid (HCl). The residues were freeze-dried and are hereafter referred to as lignin isolates. Lignin isolates were analyzed with 2D-HSQC-NMR spectroscopy, ^31^P NMR spectroscopy, size exclusion chromatography, and an antioxidant capacity assay.

### Sequential Solvent Fractionation of Brown-Rot Decayed Spruce and Birch

Bown-rot decayed wood was sequentially extracted with ethyl acetate (EtOAc), ethanol (EtOH) and 80% (v/v) acetone in water (Ace/H_2_O). Hereto, 500 mg of material was dispersed in 10 mL of EtOAc, vortexed for 30 s and head-overtail mixed at 20 rpm for 1 h. Insoluble material was separated by centrifugation (2500x*g*, 5 min, 20 °C) and mixed with 10 mL of EtOH, followed by identical extraction and separation steps, and this procedure was then repeated once more for Ace/H_2_O extraction. The ultimate residue was washed with 2 mL Ace/H_2_O, and that washing solution was combined with the Ace/H_2_O extract. All samples were dried under nitrogen atmosphere at 40 °C. The lignin extracted with 80% (v/v) acetone in water was analyzed with 2D-HSQC-NMR spectroscopy and size exclusion chromatography.

### Brown-Rot Decayed Spruce and Birch Lignin Acetylation

Lignin isolates obtained from brown-rot decayed spruce and birch (50 mg) were mixed with 2 mL pyridine/acetic anhydride (1:1 v/v), briefly vortexed and magnetically stirred for 5 h at room temperature. To precipitate the lignin, the solutions were added to 40 mL of Milli-Q water, left to set for 30 min at 4 °C, centrifuged (4700x*g*, 5 min, 20 °C), after which the pellets were washed twice with 40 mL of Milli-Q water acidified to pH 2.0 with HCl. The residues were dried on air at room temperature and analyzed with 2D-HSQC-NMR spectroscopy.

### 2D-HSQC NMR Spectroscopy

For sound and brown-rot decayed wood HSQC NMR measurements, approximately 60 mg of material (lignin isolates, acetylated lignin isolates and lignin extracted with 80% (v/v) acetone in water) was mixed with 0.6 mL DMSO-*d*_6_ in the NMR tube to form a gel, and sonicated for up to 2 h. Solution-state HSQC NMR measurements were done with approximately 30 mg of lignin isolate or acetone/H_2_O extract, again dissolved in 0.6 mL DMSO-*d*_6_. Acetylated lignin isolates were likewise dissolved in DMSO-*d*_6_ (30 mg in 0.6 mL) with the addition of 50 μL CDCl_3_ to improve dissolution.

Measurements were performed on a Bruker AVANCE III 600 MHz NMR spectrometer (Bruker BioSpin, Rheinstetten, Germany) equipped with a 5 mm cryo-probe located at MAGNEFY (MAGNEtic resonance research FacilitY, Wageningen, The Netherlands). ^1^H–^13^C HSQC spectra were recorded by using the adiabatic “hsqcetgpsisp2.2” pulse sequence using the following parameters: spectral width of 7,200 Hz (12 ppm) in F1 (^1^H) using 4096 increments for an acquisition time of 0.29 s and interscan delay of 1.0 s and a spectral width of 33,000 Hz (220 ppm) in F2 (^13^C) using 512 increments with an acquisition time of 8 ms with 16 scans per increment. The ^1^*J*_CH_ used was 145 Hz. Processing used Gaussian apodization (Gaussian broadening = 0.001, Line broadening = −0.2) in the ^1^H dimension and a squared cosine function (Squared sine bell = 2) in the ^13^C dimension. In all spectra, the central solvent peak was used as an internal reference (δ_C_ 39.5 ppm; δ_H_ 2.49 ppm). The spectra were processed using TopSpin 4.0 software.

Semiquantitative analysis of the HSQC volume integrals was performed according to Del Río et al.,^41^ making use of the chemical shifts reported in the literature for annotation.^42^ S_2,6_, G_2_ and MC_6_, C_2_ signals were used for S, G, MC (methoxylated catechol), and C (catechol) units, respectively, where S units were adjusted by halving. Oxidized analogues were estimated in a similar manner. In the aliphatic oxygenated region, β-*O*-4 aryl ether substructures and their C_α_-oxidized analogues were estimated from their C_β_-H_β_ correlations. For β-5 phenylcoumaran, β–β resinol and β-1/α-*O*-α spirodienone and arylglycerol substructures, their respective C_α_-H_α_ correlations were used. Volume integrals for β–β resinol substructures were adjusted by halving. Cinnamyl alcohol and dihydroxypropiovanillone/syringone substructures were estimated from their C_γ_-H_γ_ correlations and volume integrals were halved. In the aldehyde region, cinnamaldehyde and benzaldehyde substructures were estimated from their respective C_γ_-H_γ_ and C_α_-H_α_ correlations. Volume integration of all signals was performed at equal contour levels, with the integrals normalized to the size of the – OCH_3_ signal. The abundance of each lignin unit was then calculated as a percentage of the total lignin content, which includes G + G_ox_ + S + S_ox_+MC+MC_ox_+C. The percentage abundance was expressed per 100 aromatic rings.

### ^31^P NMR Spectroscopy

^31^P NMR was performed as previously described.^43^ Approximately 30 mg of lignin isolate was mixed with 100 μL *N*,*N*-dimethylformamide (DMF)/pyridine (50:50 v/v) and 100 μL pyridine containing 15 mg mL^–1^ cyclohexanol as internal standard and 2.5 mg mL^–1^ chromium(III) acetylacetonate as relaxation agent, and stirred overnight to dissolve. Derivatization of the dissolved lignins was performed by the addition of 2-chloro-4,4,5,5-tetramethyl-1,3,2-dioxaphopholane (100 μL premixed with 400 μL of deuterated chloroform). The phosphitylated lignins were analyzed on a Bruker AVANCE III 400 MHz instrument using a standard phosphorus pulse sequence with 30° pulse angle (“zgig30”), inverse gated proton decoupling, using 64k increments with an acquisition time of 0.67 s and interscan delay of 5 s with 256 scans per increment. The data were processed using exponential apodization with a line broadening factor of 4 Hz. Signals were assigned according to Granata and Agyropoulos (1995)^44^ and integrated by using the MestReNova 10 software (Mestrelab Research).

### Size-Exclusion Chromatography (SEC)

Alkaline SEC was performed as described by Constant et al. (Method D).^45^ Briefly, lignin was dissolved in 0.5 M NaOH (eluent) in a concentration of 1 g L^–1^ and separated by using two TSKgel GMPWxl columns (7.8 × 300 mm, particle size 13 μm) in series equipped with a TSKgel guard column PWxl (6.0 × 40 mm, particle size 12 μm). Absorption was monitored at 280 nm. Sodium polystyrenesulfonate (PSS) standards and phenol were used for calibration. Protobind 1000 lignin (Wheat straw/Sarkanda grass soda lignin, GreenValue S.A, Switzerland) was used as standard.

### Antioxidant Capacity Assay

The antioxidant capacity of lignin isolates of sound wood and brown-rot decayed wood was evaluated in a 2,2-diphenyl-1-picrylhydrazyl assay according to Rumpf et al.^46^ Hereto, 10 mg of lignin was dissolved in 2 mL 90% (v/v) aqueous dioxane and 0.1 mL of this sample solution was mixed with 3.9 mL 2,2-diphenyl-1-picrylhydrazyl solution (60 μM in 90% (v/v) aqueous dioxane). Absorption was measured at 518 nm after 30 min and calibrated against six Trolox standards in the range of 0–230 mg L^–1^. The Trolox equivalent antioxidant capacity (TEAC) was calculated as described by Rumpf et al.^46^

## Results and Discussion

### Carbohydrate and Lignin Removal

The wood decaying properties of the brown-rot fungus *G. trabeum* were examined using two lignocellulosic substrates, spruce and birch, representing fundamentally different lignin and hemicellulose structures. Extensive fungal growth of *G. trabeum* was observed on both substrates, evidenced by the well-developed mycelium throughout the wood blocks (Figure S1). Following 18 weeks of fungal growth, considerable mass loss was observed, with reductions of 69.0 ± 0.3% and 68 ± 2% in spruce and birch, respectively. Notably, mass loss plateaued after 12 weeks, indicating that the 18-week samples used for detailed lignin structure analysis represented the end stage of wood conversion by *G. trabeum* (Figure S2).

**Figure 1:**
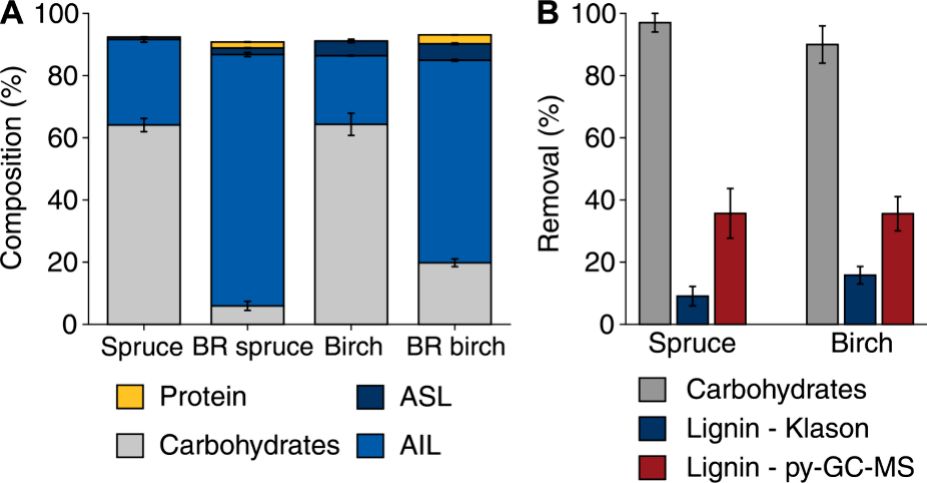
**Compositional analysis of spruce and birch.** (A) The content of protein, acid soluble lignin (ASL), acid insoluble lignin (AIL), and carbohydrates in spruce and birch, as percentage of the total mass, determined before and after 18 weeks of brown-rot (BR) decay. (B) Removal of carbohydrates and lignin from spruce and birch after brown-rot decay, calculated based on contents analyzed (w/w%; Table S3) combined with the total gravimetric dry mass removal. Lignin was quantified with gravimetric Klason methodology (Lignin - Klason; sum of ASL and AIL) and ^13^C-IS pyrolysis-GC-MS (Lignin - py-GC-MS). Error bars represent standard deviation (*n* = 3).

The changes in the wood composition before and after fungal treatment were assessed using gravimetric and compositional analyses (Figure 1A and Table S3–S4). These analyses revealed extensive depletion of carbohydrates (>90%), accompanied by a higher lignin content per dry matter (w/w%), in the decayed wood samples. These results clearly show preferential degradation and metabolization of polysaccharides over lignin and is characteristic for wood decay by brown-rot fungi.^21,47,48^

The proportion of lignin expectedly increased in the brown-rot decayed wood due to preferential removal of carbohydrates. Still, our data (Figure 1B) indicate that *G. trabeum* also removed some lignin, as has been observed by others.^20−22^ Accurately quantifying lignin in treated lignocellulosic biomass samples remains challenging due to limitations of traditional analytical methods. The Klason method, while widely used, suffers from methodological limitations, due to its gravimetric and unselective nature, as also discussed by others.^26−28^ Additionally, some residual fungal mycelium inevitably remains in the decayed wood. The identified nitrogen (Table S3 & S4) in the decayed wood samples may originate from proteins and/or the cell wall of the fungus in the form of chitin, which may infer with Klason lignin quantification. To address this issue, we additionally quantified the lignin content using a recently developed approach utilizing ^13^C-IS pyrolysis-GC-MS.^30^ The determined lignin contents of sound spruce and birch wood were not different for the different analytical method used (Klason vs ^13^C-IS pyrolysis-GC-MS; Figure S3), aligning with earlier findings.^39^ However, discrepancies emerged when analyzing the decayed wood, where the Klason method estimated a substantially higher lignin content (83% for spruce and 70% for birch) compared to the ^13^C-IS pyrolysis-GC-MS method (55% for spruce and 59% for birch) (Figure S3). Given the fact that the pyrolysis-GC-MS method relies on the sum of all lignin-derived pyrolysis products (Table S1), we first checked whether any new products were formed upon brown-rot action, potentially underlying the lower lignin contents found with ^13^C-IS py-GC-MS. Indeed, several pyrolysis products tentatively annotated as catechols and methoxycatechols were detected (Figure S4 & Table S2), in line with previous reports on the *O*-demethylation activity of brown-rot fungi.^20,21,23^ By accounting for catechols and methoxycatechols a slight increase in lignin content was obtained (Figure S3). However, the estimated lignin removal still remained higher using the py-GC-MS method (36% for both spruce and birch) compared to the Klason method (9% and 16% for spruce and birch, respectively) (Figure 1B). These observations could suggest potential shortcomings in both methods. Inaccurate relative response factors for *O*-demethylated pyrolysis products might lead to underestimation of lignin contents (and, thus, overestimation of removal) when using the ^13^C-IS py-GC-MS method, highlighting the need for further refinement of the method. Conversely, the Klason method likely overestimated the lignin content of brown-rot decayed wood (and, thus, underestimation of removal) due to its inability to distinguish residual fungal constituents from lignin in the acid-insoluble residue.

The actual amount of lignin removal by *G. trabeum* is therefore likely in-between the removal values obtained from the two methods (Figure 1B). Importantly, regardless of the method used, our results establish that lignin removal from both softwood and hardwood is substantial, challenging the notion that lignin removal by brown-rot fungi is negligible.^47^ The fate of this lignin removed from the wood remains unclear and requires further investigation. One possibility is that the lignin was deposited into the surrounding soil in the experimental setup (Figure S1A). Another possibility is that the lignin was metabolized by the fungus as recently shown for *Agaricus bisporus*.^49^

### Structural Characterization of *G. trabeum* Decayed Wood

Building on the insights from the ^13^C-IS py-GC-MS analysis of lignin content and delignification, we aimed to further utilize this method to investigate structural changes in the remaining lignin after brown-rot decay. To achieve this, comparative characterization of lignin in sound and brown-rot decayed wood was performed, specifically quantifying lignin specific pyrolysis products indicative of oxidative modifications. This focus on oxidative modifications is crucial since brown-rot fungi primarily degrade wood through oxidative processes.

#### ^13^C-IS py-GC-MS Analyses Indicate Extensive Oxidation and Demethylation

The ^13^C-IS py-GC-MS analysis showed that the lignin in sound spruce is dominated by G-units, with traces of H- and S-units, while sound birch contains S and G units, with minor amounts of H units (Table 1), well aligned with the typical lignin structures found in softwood and hardwood species.^6^ The subunit composition remained unchanged in brown-rot decayed spruce and birch, indicating that *G. trabeum* did not selectively remove one unit over the other. Nonetheless, substantial C_α_-oxidation could be observed in both wood types. In line with the more extensive accumulation of lignin, C_α_-oxidized moieties increased 1.9-fold in spruce and 1.5-fold in birch (Table 1).

**Table 1:** Relative Abundance of Lignin-Specific^13^C-IS py-GC-MS Pyrolysis Products in Sound and Brown-Rot Decayed (BR) Wood.^a^

Lignin subunits (%)	Sound spruce	BR spruce	Sound birch	BR birch
H	1.3 ± 0.1	1.3 ± 0.0	0.9 ± 0.1	0.7 ± 0.0
G	97.1 ± 0.3	98.4 ± 0.0	25.7 ± 1.0	25.9 ± 0.2
S	1.6 ± 0.4	0.2 ± 0.0	73.4 ± 1.1	73.5 ± 0.2
Structural moieties (%)				
Unsubstituted	5.8 ± 0.1	7.0 ± 0.2	3.9 ± 0.1	4.8 ± 0.5
Methyl	5.0 ± 0.2	4.6 ± 0.1	2.6 ± 0.1	2.0 ± 0.1
Vinyl	15.2 ± 0.2	15.5 ± 0.5	11.0 ± 0.2	8.7 ± 0.7
C_α_ – ox^b^	4.7 ± 0.1	9.0 ± 0.3	6.5 ± 0.0	10.0 ± 0.5
Vanillin	2.3 ± 0.1	4.6 ± 0.1	0.7 ± 0.0	1.0 ± 0.0
Acetovanillone	1.1 ± 0.0	1.4 ± 0.0	0.3 ± 0.0	0.4 ± 0.0
Guaiacyl diketone	0.6 ± 0.0	2.1 ± 0.1	0.2 ± 0.0	0.6 ± 0.0
Syringaldehyde	0.1 ± 0.0	n.d.	3.0 ± 0.0	3.8 ± 0.2
Acetosyringone	n.d.	n.d.	1.0 ± 0.0	1.3 ± 0.1
Syringyl diketone	n.d.	n.d.	0.8 ± 0.0	1.8 ± 0.1
C_β_ – ox^b^	2.3 ± 0.0	3.1 ± 0.0	2.0 ± 0.2	2.2 ± 0.1
C_γ_ – ox^b^	61.8 ± 0.4	56.9 ± 1.2	69.0 ± 0.3	68.5 ± 2.1
Miscellaneous	5.3 ± 0.1	3.9 ± 0.1	5.1 ± 0.1	3.8 ± 0.3
PhC_γ_^c^	68.6 ± 0.4	64.2 ± 1.0	76.4 ± 0.3	76.4 ± 1.7
PhC_γ_ -diketones^d^	68.4 ± 0.4	63.4 ± 1.0	76.2 ± 0.3	75.8 ± 1.7

a For a comprehensive list of all monitored pyrolysis products and their respective categories, see Table S1. Numbers are averages of triplicates with standard deviation. Standard deviations <0.05 are reported as 0.0 n.d: not detected.

b Pyrolysis products oxidized on carbon α, β, or γ.

c Pyrolysis products with an intact α, β, γ – carbon side chain. “Ph” refers to the phenyl group (C_6_H_5_).

d PhC_γ_ with diketones excluded.

Notably, pyrolysis products of aldehydes (vanillin and syringaldehyde), ketones (acetovanillone and acetosyringone), and diketones (guaiacyl diketone, syringyl diketone) all increased substantially. This suggests the presence of oxidized lignin substructures within the brown-rot decayed wood, resulting from various underlying ligninolysis mechanisms, as previously demonstrated.^31,50^ The relative increase in C_α_-oxidized pyrolysis-products in brown rot decayed spruce was accompanied by a slight decrease in three-carbon side chain (PhC_γ_) pyrolysis products in spruce, which is indicative of interunit bond cleavage in the lignin polymer.^31,51^ As discussed previously in studies of white-rot and *Agaricus bisporus* treated lignin, the abundance of PhC_γ_ products serves as a measure of intact interunit linkages because these pyrolysis products only arise from uncleaved three-carbon side chains. Combining these observations, the decrease in PhC_γ_ products suggests that some of the formed C_α_-oxidized moieties may originate from oxidative cleavage of intact lignin interunit linkages. In addition to the extensive oxidation observed, eight pyrolysis products were identified in the brown-rot decayed spruce and birch that were tentatively annotated as either catechol, methoxy catechol, or their derivatives (Figure S4; Table S2). Consequently, the detection of these catechol and methoxy catechol moieties is a clear indication that significant net *O*-demethylation occurred.

#### HSQC NMR Analysis Confirms the Results Obtained with ^13^C-IS py-GC-MS

**Figure 2:**
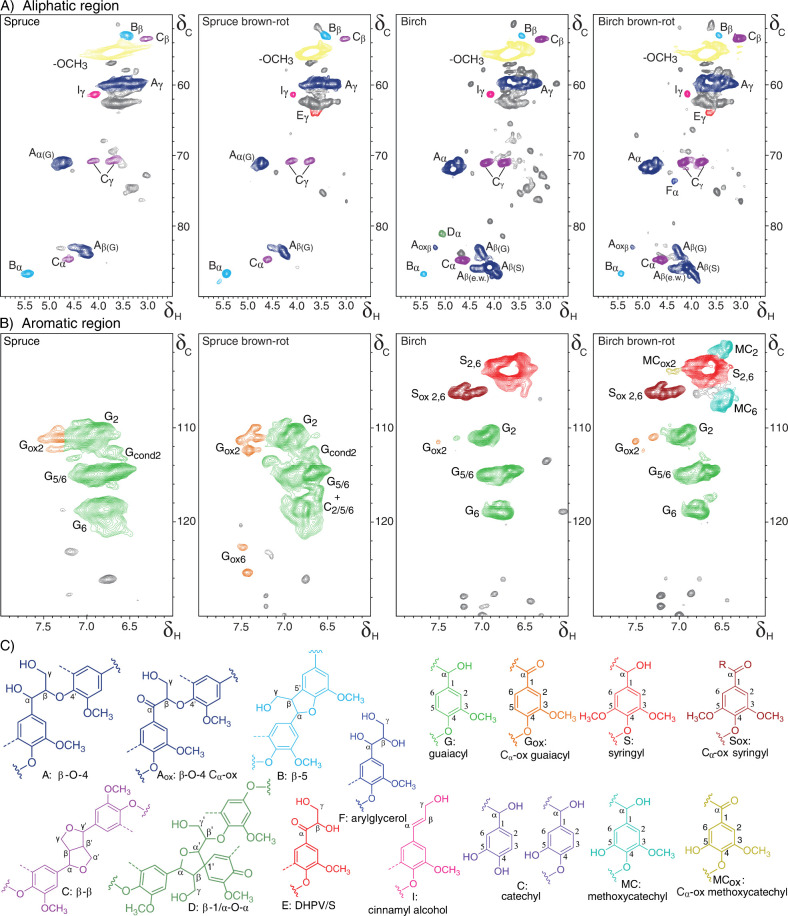
**HSQC NMR spectra of lignin isolates of sound and brown-rot decayed wood.** The spectra show the aliphatic (A) and aromatic (B) regions for lignin isolated (through enzymatic treatment) from sound and brown-rot decayed spruce and birch. Subscripted numbers and Greek letters in annotations indicate which carbon in the annotated substructure the signal originates from. (C) Annotated substructures, where colors correspond to colored signals in A and B. Dashed lines indicate -H (guaiacyl) or -OCH_3_(syringyl), while the main position for further coupling is indicated with wavy lines. Unassigned peaks are shown in gray.

To substantiate the ^13^C-IS py-GC-MS observations, we analyzed HSQC NMR spectra of sound and brown-rot decayed wood (Figure S5). The aromatic regions confirmed the presence of increased levels of oxidized moieties in the brown-rot decayed wood. Expectedly, the aliphatic regions of these spectra were unsuitable for detailed analysis due to significant overlap between signals from lignin and carbohydrates (data not shown). Therefore, to enable detailed characterization of the aromatic and aliphatic regions of the lignin, carbohydrates were enzymatically removed, and the resulting lignin isolates were subjected to HSQC NMR analysis. The spectra of the lignin isolates showed clear differences between sound and brown-rot decayed wood (Figure 2), which were further materialized by semiquantitative analysis of the volume integrals (Table 2).

**Table 2:** Semi-Quantitative HSQC NMR Characterization of Lignin Isolated from Sound and Brown-Rot Decayed Wood.^a^

Subunits (%)	Sound spruce	BR spruce	Sound birch	BR birch
G	87.6	75.1	22.5	23.5
G_ox_	4.6	10.3	0.3	0.8
G_cond_	7.9	14.6	0.0	0.0
S	0.0	0.0	69.1	54.0
S_ox_	0.0	0.0	8.1	10.8
MC	0.0	0.0	0.0	9.2
MC_ox_	0.0	0.0	0.0	1.7
S/G	-	-	3.4	2.7
Interunit linkages (per 100 ar)				
β-*O*-4 aryl ether	30.6	30.3	62.3	49.6
β-5 phenylcoumaran	8.6	10.0	1.7	1.8
β–β resinol	2.8	2.7	5.7	6.9
β-1 spirodienone	0.0	0.0	2.2	0.0
5–5/4-*O*-β dibenzodioxocin	2.2	0.8^b^	0.0	0.0
End units (per 100 ar)				
Cinnamyl alcohol	3.1	1.3	1.7	1.3
Cinnamaldehyde	5.1	3.5	2.0	2.9
Arylglycerol	0.0	0.0	0.0	2.3
Benzaldehyde	2.0	6.3	0.3	1.6
HPV/HPS	0.8	0.9	0.3	0.7
DHPV/DHPS	0.9	3.3	0.0	1.4
Ring substituents (per 100 ar)				
Methoxyl	134.6 (127.5)^c^	122.1(103.4)^c^	183.2	167.2

a G_ox/_S_ox_: guaiacyl/syringyl units oxidized on the C_α_-carbon, G_cond_: guaiacyl units involved in condensed linkages, MC: methoxycatechyl units, MC_ox_: methoxycatechyl units oxidized on the C_α_-carbon,HPV/HPS: hydroxypropiovanillone/hydroxypropiosyringone,DHPV/DHPS: dihydroxypropiovanillone/dihydroxypropiosyringone. “ar” refers to aromatic rings

b Integrated at 2x zoomed counter level.

c Values in parentheses based on total aromatic region to account for C_2_, C_5_, C_6_, G_5_, G_6_ overlap.

**Figure 3:**
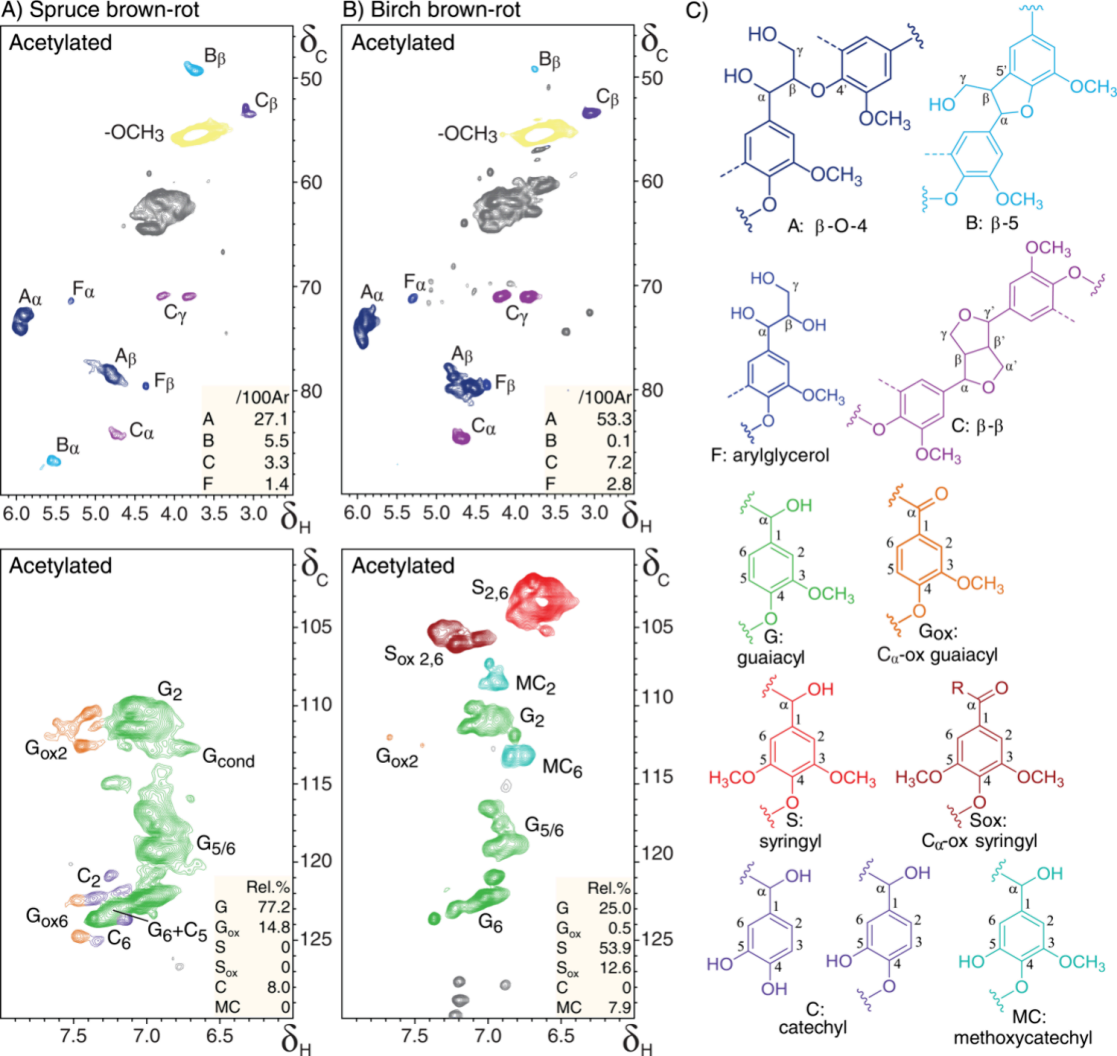
**HSQC NMR spectra of acetylated lignin isolates of brown-rot decayed wood.** The spectra show the aliphatic and aromatic regions for acetylated, brown-rot decayed spruce (A) and birch (B). Subscripted numbers and Greek letters in annotations indicate which carbon in the annotated substructure the signal originates from. Inset values present lignin component ratios determined from contour volume integrals (top: per 100 aromatic rings, bottom: relative). (C) Annotated substructures, where colors correspond to colored signals in the spectra. Dashed lines indicate -H (guaiacyl) or -OCH_3_ (syringyl), while the main position for further coupling is indicated with wavy lines. Unassigned peaks are shown in gray.

The spectra showed a clear increase in C_α_-oxidized subunits in brown-rot decayed wood and formation of methoxycatechols in brown-rot decayed birch, confirming observations from ^13^C-IS py-GC-MS. The absence of methoxycatechols in the brown-rot decayed softwood indicated that the aromatic rings have probably not undergone hydroxylation. Therefore, the formation of methoxycatechol in brown-rot decayed birch, is more likely to be caused by net *O*-demethylation of S-units, rather than hydroxylation of G-units. This notion is further corroborated by the substantial decrease in methoxyl abundance (Table 2). Conversely to methoxycatechol units, which can readily be distinguished from other signals (Figure 2B), signals from catechol moieties overlap with guaiacyl unit signals.^52^ Brown-rot decayed spruce also showed a substantial decrease of methoxyl content (Table 2), suggesting the presence of such catechols after brown-rot decay of this G-rich material. To be able to discriminate catechol and guaiacyl moieties, the lignin isolates of brown-rot decayed wood were acetylated. Acetylation derivatizes the existing hydroxyl groups on the aromatic rings, enabling differentiation between guaiacyl and catechol moieties (Figure 3A).^53^ The spectra of the acetylated lignins indeed showed the expected presence of catechols. The spectra also confirmed the presence of methoxycatechols in brown-rot decayed birch lignin, and as well as the absence of catechols, which confirms the ^13^C-IS py-GC-MS data and shows that *O*-demethylation of syringyl units was preferred over *O*-demethylation of guaiacyl units. Interestingly, both brown-rot decayed wood substrates showed comparable levels of *net* demethylated units (approximately 8% of the total subunits present). A decrease in methoxyl content is in line with previous research on brown-rot decayed wood.^21^

In line with the net demethylation observed, ^31^P NMR analysis of lignin isolates of sound and brown-rot decayed wood showed an overall increase in phenolic OH content, with a 2.3 and 1.7-fold increase in spruce and birch, respectively (Figure S6, Table S5). For both spruce and birch, an increased signal at 137.5–138.5 ppm (Figure S6) further evidence the occurrence of (methoxy)catechol substructures.^54^ The ^31^P NMR analysis also demonstrated a substantial increase in carboxylic acid moieties, indicating that part of the increase in C_α_-ox units was due to benzoic acid substructures being formed.

#### Interunit Oxidative Cleavage Occurs in β-O-4 Aryl Ethers

The detection of oxidized moieties in the aromatic region of the NMR spectra (Figure 2B), aligning with the detection of oxidized products by ^13^C-IS py-GC-MS, prompted us to investigate the origin of these oxidized moieties. Specifically, we aimed to investigate if some originated from oxidative cleavage of interunit linkages within the lignin polymer. To address this question, we analyzed the aliphatic region of the NMR spectra (Figure 2A) complemented with the relative abundance of oxidized pyrolysis products. The aliphatic region in the acetylated NMR spectra revealed a reduction in β-*O-*4 aryl linkages in both spruce (11% reduction) and birch wood (14% reduction) after brown-rot decay. β-1 spirodienones were undetectable in sound and brown-rot decayed spruce, while they disappeared completely in birch after brown-rot decay. In contrast, the more resistant β–β resinol and β-5 phenylcoumaran linkages remained unchanged in both wood types. This suggested that cleavage of interunit linkages by *G. trabeum* in hardwood and softwood primarily occurs in β-*O-*4 aryl ethers. Despite the cleavage of interunit linkages, SEC analysis showed that the lignin in brown-rot decayed wood remained of high molecular weight, with a bimodal distribution (Figure S7 & Table S6). The HSQC NMR spectra of acetylated lignins, and ^31^P NMR spectra confirmed that phenolic end groups remained and accumulated. Therefore, repolymerization of the lignin structures is considered unlikely to have occurred, at least not extensively.

Identification of cleaved β-*O-*4 aryl ethers prompted an investigation into which specific bonds within the β-*O-*4 aryl linkage were cleaved. The NMR spectra offer valuable information about end units and when complemented by information provided by diagnostic substructures observed with ^13^C-IS py-GC-MS, these data can shed light on the cleavage pattern. It is important to keep in mind that a significant portion of the lignin was removed by the fungus (Figure 1B), hence, the analysis of the cleavage pattern is necessarily limited to the lignin fraction that was not removed.

One potential cleavage site in the β-*O-*4 aryl ether linkage is the C_α_-C_β_ bond, where cleavage would produce a benzaldehyde end unit, and, upon further oxidation, benzoic acid moieties. Structural lignin analysis with NMR (Table 2) revealed a substantial increase in benzaldehyde units in both spruce (from 2.0% to 5.9%) and birch (from 0.3% to 1.6%). Consistent with this, ^13^C-IS py-GC-MS analysis (Table 1) showed an increase in benzaldehyde-derived pyrolysis products, including vanillin and syringaldehyde. Additionally, some of the formed benzaldehydes can be oxidized to benzoic acids due to the oxidizing environment created by the fungus. ^31^P NMR analysis (Figure S6; Table S5) confirmed an increase in benzoic acid structures in both brown-rot decayed woods, underpinning that C_α_-C_β_ cleavage indeed had occurred.

Cleavage at the *O*-4 position would result in the formation of arylglycerol end units,^55^ which can be further oxidized to dihydroxypropiovanillone (DHPV) and dihydroxypropiosyringone (DHPS) substructures. Spruce exhibited a more than 3-fold increase in DHPV after brown rot decay, while birch showed an increase in both arylglycerols and DHPV/DHPS (Table 2). In accordance, ^13^C-IS py-GC-MS analysis showed an accumulation of guaiacyl diketones (spruce) and syringyl diketones (birch), which have previously been identified as diagnostic markers for *O*-4 cleavage of β-*O-*4 bonds due to their origin from DHPV/DHPS.^31^ This data therefore strongly suggests that *O*-4 cleavage was also a cleavage pathway for both substrates.

While less common than C_α_-C_β_ and *O*-4 cleavage, cleavage of the β-O bond could occur, leading to the formation of hydroxypropiovanillone (HPV) structures. Spruce samples showed no significant change in HPV content, while birch exhibited a slight increase. Thus, this cleavage route appears to be a minor contributor to the β-*O-*4 aryl cleavage in both spruce and birch.

Overall, these findings therefore suggest that the structural modifications and more specific β-*O-*4 aryl ether cleavage routes are the same for spruce and birch, and thus seem not majorly influenced and driven by the lignin structure itself. Since *G. trabeum* does not express lignin-degrading peroxidases, the bond cleavages are likely the result of oxidation by hydroxyl radicals generated through Fenton chemistry.

### Valorization Potential of Brown-Rot Decayed Lignin

The ability of brown-rot fungi to selectively target carbohydrates showcases their potential as a promising biotechnological system for improved valorization of lignin. Recently, Wu et al. highlighted how *O*-demethylation has emerged as a strategy to enhance lignin reactivity, enabling its use in catalytic processes and the development of valuable products.^56^ Therefore, the presence of accumulating catechols and methoxycatechols along with an increased overall phenolic content following *O*-demethylation and interunit linkage cleavage demonstrated herein, positions brown-rot decayed hardwood and softwood as a potential valuable resource.

To further assess the potential of brown-rot fungi for improved properties of lignin, we conducted an assay to evaluate if the increased phenolic content of the lignin translates into a higher antioxidant capacity (Figure S8). This revealed that brown-rot decayed lignin possesses a greater antioxidant capacity than lignin in sound wood, emphasizing its higher reactivity, which is relevant for oxidoreductases relying on reducing agents^57,58^ as well as for lignin valorization strategies targeting antioxidant properties.^59^ Given the higher antioxidant capacity of birch lignin compared to spruce lignin, as expected based on its methoxy-rich structure, brown-rot decayed hardwood lignin seems most promising for these application directions. Conversely, the abundance of phenolic guaiacyl and catechyl units makes brown-rot decayed softwood lignin a more interesting resource for resin applications.^60^

A common step in the valorization of lignin is its separation into fractions with distinct properties. Hence, here we attempted to fractionate the brown-rot decayed lignin by sequential solvent fractionation, employing an EtOAc-EtOH-Acetone/water sequence. Conversely to lignin obtained through typical Kraft^61,62^ or deep eutectic solvent pulping,^63^ the brown-rot decayed lignins had negligible solubility in EtOAc and EtOH. Nonetheless, acetone/water extracted substantial material, varying from 18% and 25% of the brown-rotted wood dry matter for spruce and birch, respectively (Figure S9). SEC analysis of this lignin fraction confirmed a lower molecular weight nature (Figure S7), in the order of 2000 g mol^–1^, and reduced dispersity (Table S6). It would seem that this extractable fraction underlies the bimodal distribution observed for the unfractionated lignin (Figure S7). Accordingly, HSQC NMR characterization of the extracted lignin fractions revealed distinct properties (Figure S10, Table S7). The extractable lignin from brown-rot decayed spruce was enriched in C_α_-oxidized and condensed subunits, while the extractable lignin from brown-rotted birch had higher levels of methoxycatechyl and syringyl units. The extractable lignin from brown-rot decayed spruce and birch had a significantly lower methoxyl content and, despite their lower average molecular weight, retained high β-*O-*4 aryl ether content. The lower molecular weight combined with an increased (methoxy)catechol content make these lignin fractions worthwhile to explore further for the applications mentioned above.

## Conclusions

In this study we have compared wood composition and lignin structure in hardwood and softwood decayed by the brown-rot fungus *G. trabeum*. By combining HSQC NMR and ^13^C-IS py-GC-MS, we revealed that brown-rot fungi may remove more lignin from wood than previously reported in literature. The detailed structural characterization of the brown-rot decayed lignins, enabled by combination of the two analytical approaches, revealed extensive oxidation and net *O*-demethylation in both spruce and birch lignin. The results show that, despite their fundamentally different structures, spruce and birch lignin undergo remarkably similar conversions when subjected to the lignin-degrading machinery of *G. trabeum*. Consequently, the resulting lignin modifications appear to be more strongly driven by fungal capabilities rather than by the lignin structure itself.

Furthermore, we show that brown-rot decay of lignin not only yields a lignin stream enriched in phenolic substructures, with enhanced reactivity, but also allows for the straightforward separation of this lignin into distinct fractions based on molecular weight and solubility. These findings contribute to a deeper understanding of how brown-rot fungi convert lignin and highlight the broader potential of using brown-rot fungi as a strategy for lignin valorization. Specifically, approaches for valorization of lignocellulose based on brown-rot fungi offer an alternative to traditional methods by generating lignin streams that are functionally enhanced and more easily processed into valuable products. This positions brown-rot fungi as a promising tool for creating distinct and reactive lignin fractions that can be better utilized in various industrial applications.

## Supporting Information Available

Development of *G. trabeum* mycelium on wood blocks; mass loss of spruce and birch wood blocks; determination of lignin content; ^13^C-IS pyrolysis-GC-MS abundance of catechol/methoxycatechol pyrolysis products; aromatic regions of HSQC NMR spectra of sound and brown-rot decayed wood; quantitative ^31^P NMR spectra; SEC elution profiles; antioxidant capacity assay; relative mass of fractions after sequential solvent fractionation; HQSC NMR spectra of acetone/H_2_O extracts of brown-rot decayed wood; composition of carbohydrates, lignin, and protein in sound and brown-rot decayed wood; composition of structural carbohydrates in sound and brown-rot decayed wood; identity and structural classification of lignin-derived pyrolysis products detected; annotation of catechol and methoxycatechol pyrolysis products; molecular weight distribution of brown-rot decayed spruce and birch lignin isolates, and acetone/H_2_O extracts of these; structural characterization of acetone/H_2_O extracts of brown-rot decayed spruce and birch (PDF)

## Abbreviations

AIL: acid insoluble lignin
ASL: acid soluble lignin
BR: brown rot
HSQC NMR: heteronuclear single-quantum coherence nuclear magnetic resonance
MS: mass spectrometry
^13^C-IS-py-GC-MS: pyrolysis gas chromatography–mass spectrometry with uniformly, ^13^Carbon-labeled lignin as an internal standard
SEC: size-exclusion chromatography
TEAC: trolox equivalent antioxidant capacity.
