# Supplementary material for: Detailed Characterization of the Conversion of Hardwood and Softwood Lignin by a Brown-Rot Basidiomycete
Source: Biomacromolecules. 2025 Jan 6;26(2):1063–74. doi: 10.1021/acs.biomac.4c01403 (PMC11815815; doi:10.1021/acs.biomac.4c01403)
Supplement: Supplementary file 1 — bm4c01403_si_001.pdf [file bm4c01403_si_001.pdf]

Supporting information

Detailed characterization of the conversion of hardwood and softwood lignin by a brown-rot basidiomycete

Morten Rese[1§], Gijs van Erven[2,3§], Romy J. Veersma[3], Gry Alfredsen[4], Vincent G. H. Eijsink[1], Mirjam A. Kabel[3] and Tina R. Tuveng[1].*

[1]Faculty of Chemistry, Biotechnology, and Food Science, Norwegian University of Life Sciences (NMBU), P.O. Box 5003, 1433 Ås, Norway

[2]Wageningen Food and Biobased Research, Bornse Weilanden 9, 6708 WG, Wageningen, The Netherlands

[3]Wageningen University & Research, Laboratory of Food Chemistry, Bornse Weilanden 9, 6708 WG, Wageningen, The Netherlands

[4]Norwegian Institute of Bioeconomy Research, Division of Forestry and Forest Resources , P.O. Box 115, NO-1431, Ås, Norway.

*Email: tina.tuveng@nmbu.no

[§]These authors contributed equally to this study

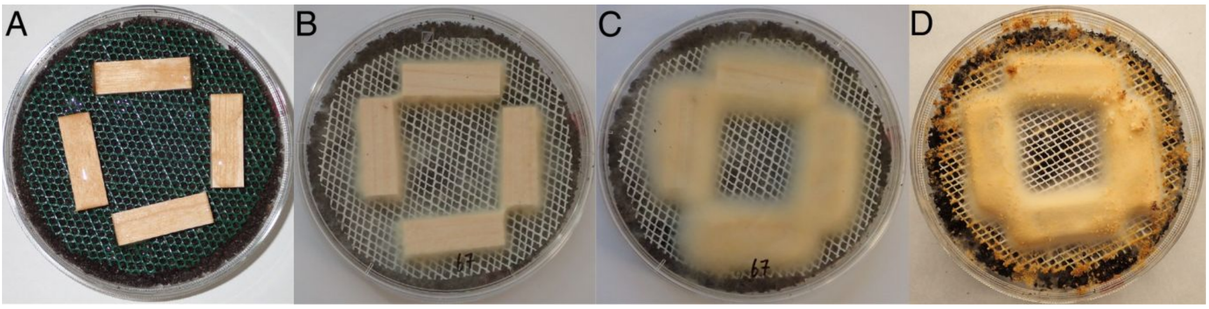

Figure S1. Development of *G. trabeum* mycelium on spruce wood blocks. (A) Wood blocks just after inoculation with liquid culture, and (B) 3 weeks, (C) 6 weeks, and (D) 18 weeks post-inoculation, i.e., at the time of harvest. The plates were incubated at 22 °C. The pictures are also representative for growth on birch.

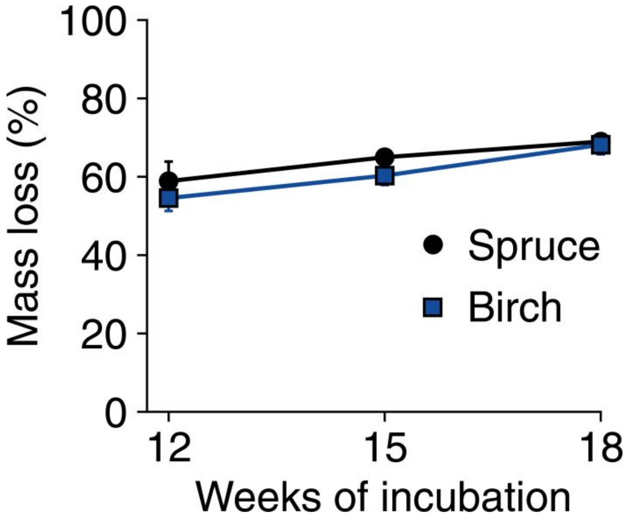

Figure S2. Mass loss of spruce and birch wood blocks during incubation with *G. trabeum*. Calculation of mass loss was done using four replicates with error bars representing standard deviation. Mass loss was calculated in percentage based on the change between initial and final dry weight.

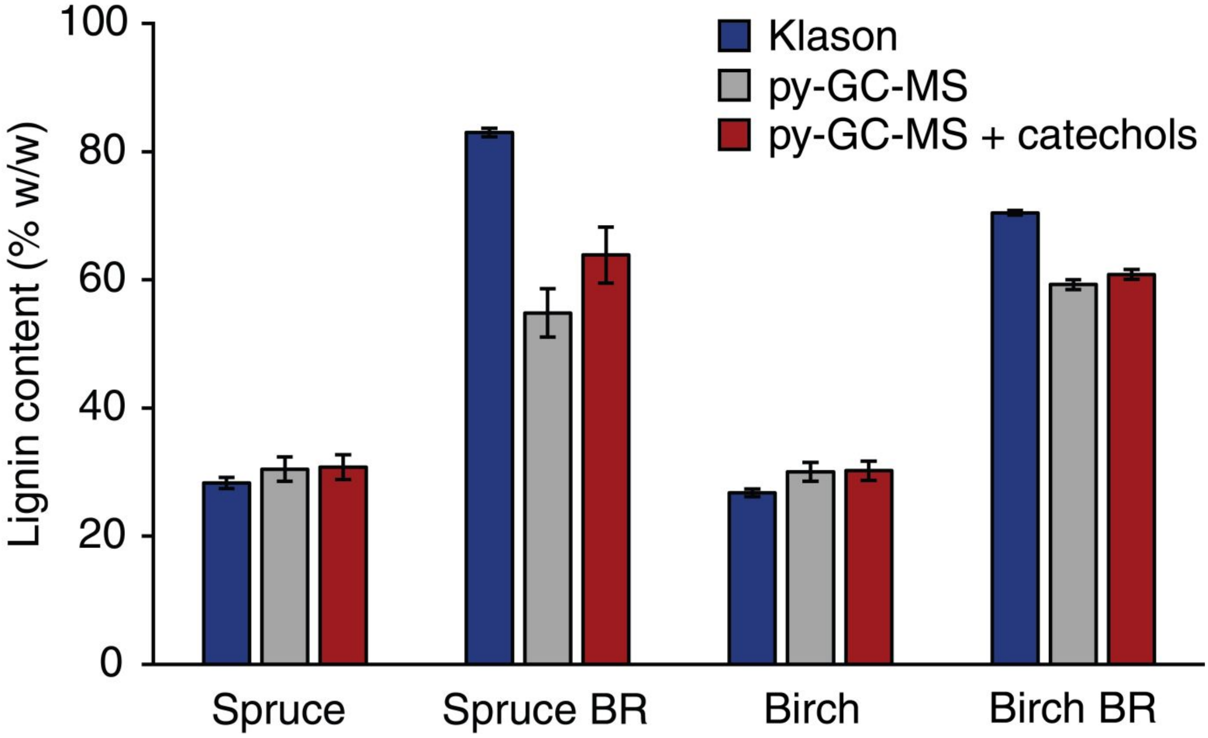

Figure S3. Lignin content in sound and brown-rot decayed wood (18 weeks decay). Determination of the lignin content was done by the Klason method blue bars), ^{13}C-IS pyrolysis-GC-MS without (grey bars) and with catechols included in the calculation (red bars). Error bars show standard deviation (n=3).

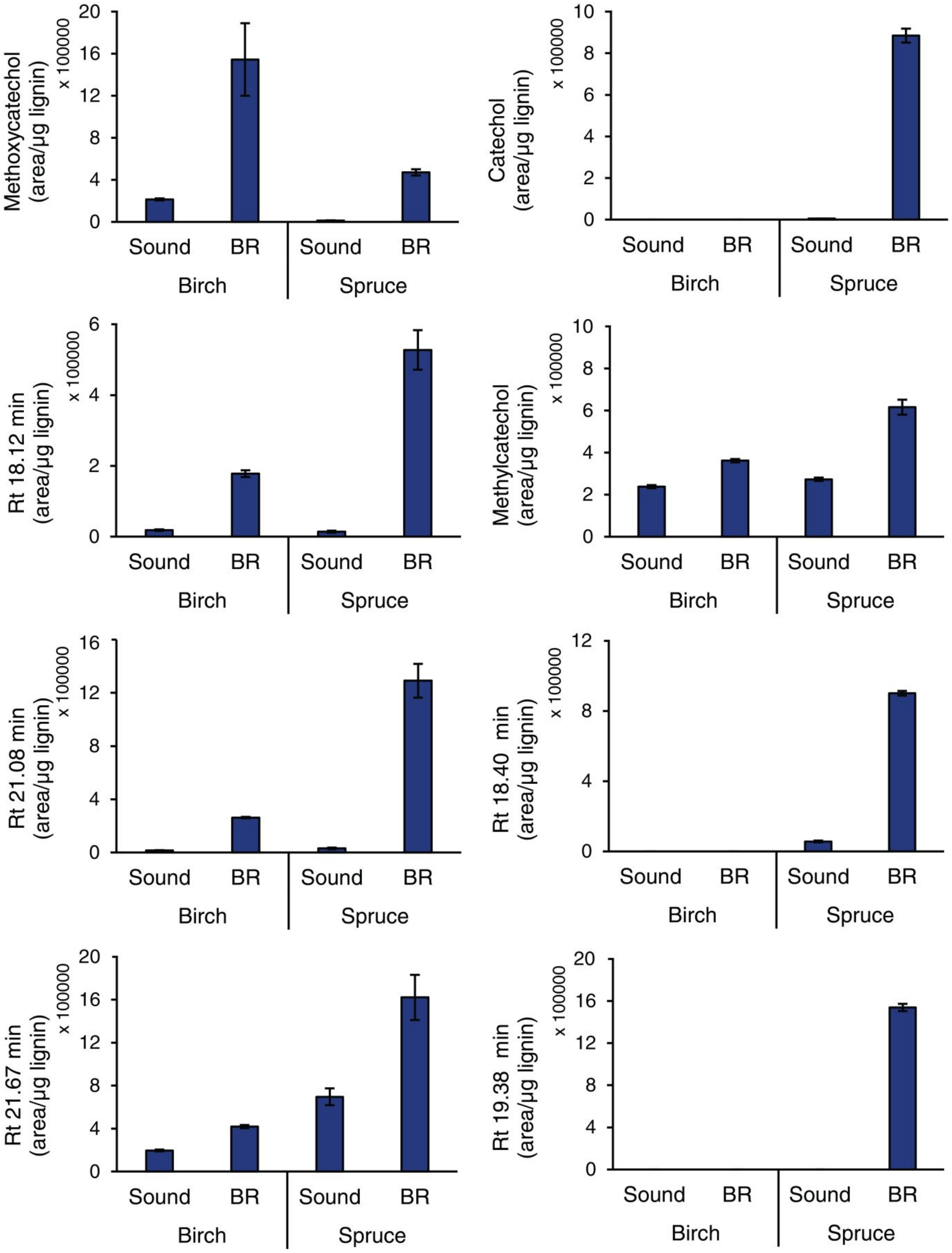

Figure S4. Abundance of catechol and methoxycatechol pyrolysis products. The graphs show the abundance of catechol/methoxycatechol pyrolysis products in sound and brown-rot decayed wood, as determined by ^{13}C-IS pyrolysis-GC-MS. See Table S2 for (tentative) annotation. The name of the compound or the retention time (for catechol and methoxycatechol derivatives) for the peak used for calculation is given on the y-axis. Error bars show standard deviation (n=3).

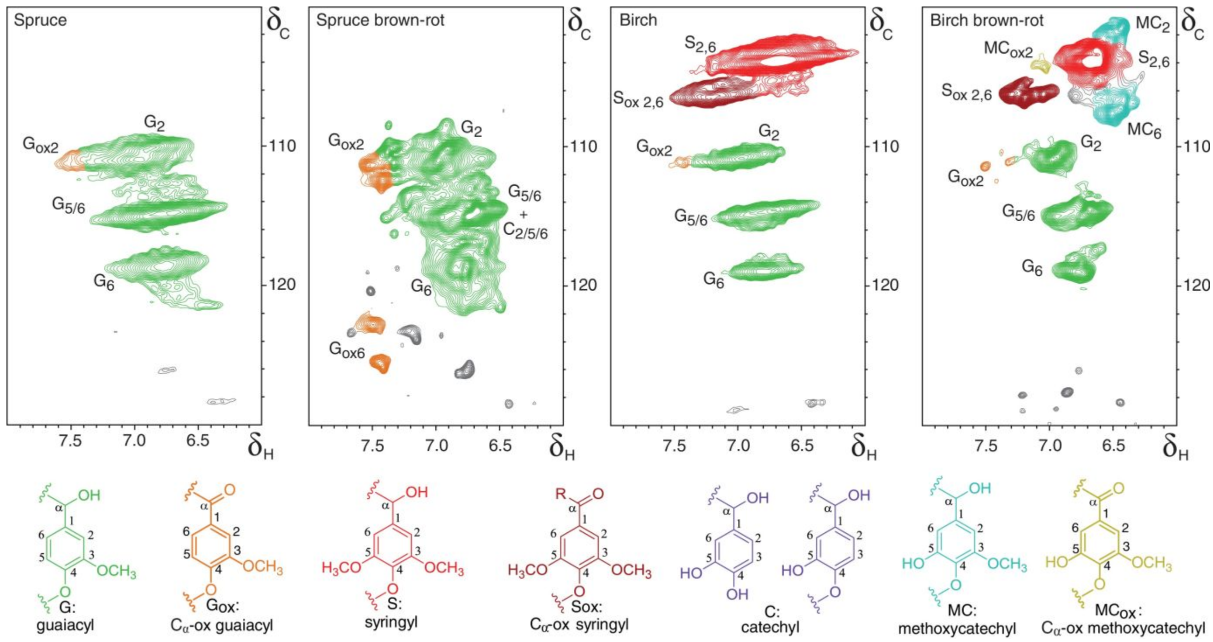

Figure S5. Aromatic regions of HSQC NMR spectra of sound and brown-rot decayed wood. Subscripted numbers and Greek letters in the annotated molecular structures indicate which carbon in the annotated substructure the signal originates from. Annotated substructures have colors corresponding to colored signals. The main position for further coupling is indicated with wavy lines. Unassigned peaks are shown in grey.

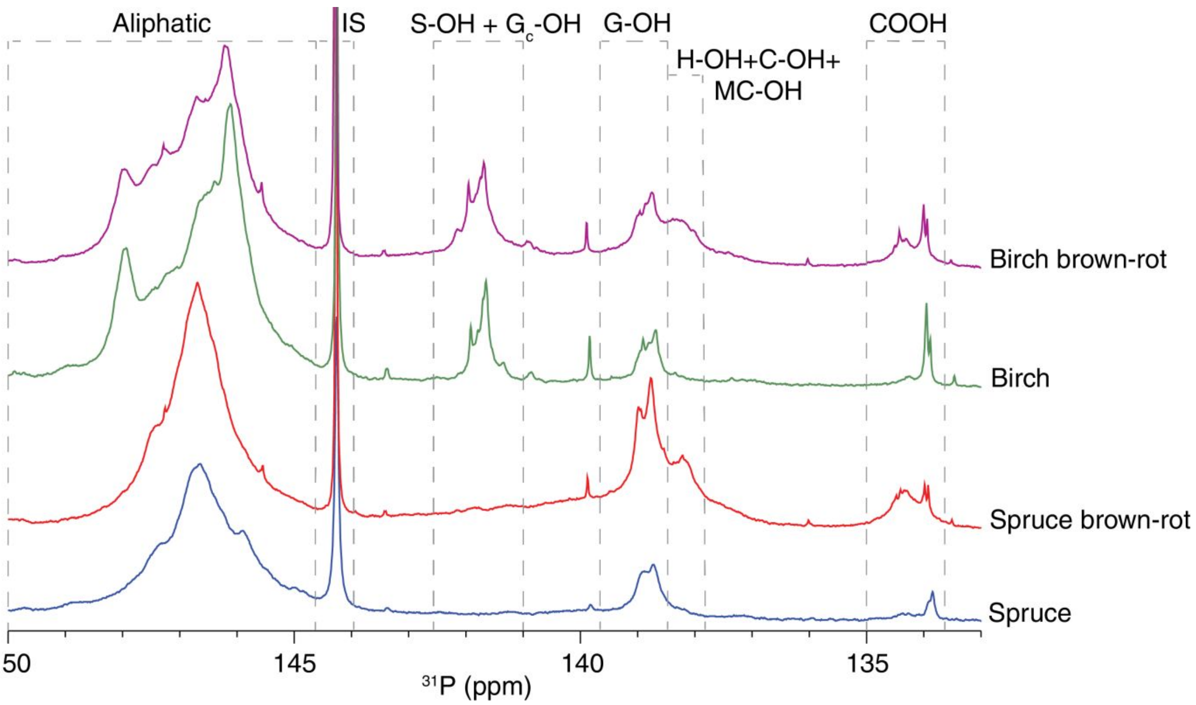

Figure S6. ^{31}P NMR spectra after phosphitylation of lignin isolated (through enzyme treatment) from sound and brown-rot decayed wood. The peaks ar annotated as aliphatic hydroxyl groups, phenolic hydroxyl groups including syringyl (S-OH) and guaiacyl (G-OH) units, carboxylic acid groups (COOH), and "H-OH + C-OH + MC-OH" represents hydroxyl groups on H-units, catechol, and methoxycatechol.

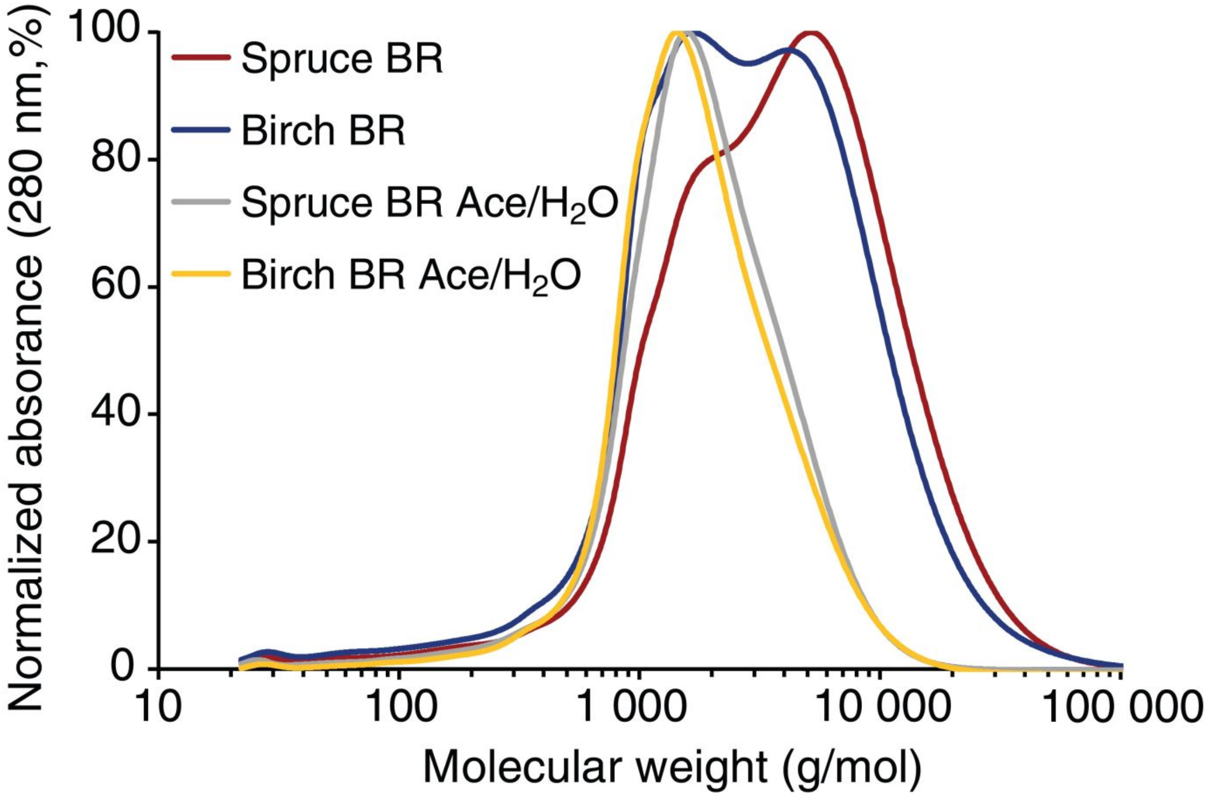

Figure S7. Alkaline SEC elution profiles of lignin isolated from brown-rot decayed wood. Lignin isolates of brown-rot decayed spruce and birch, and acetone/H_2O extracts of these, were dissolved in 0.5 M NaOH prior to SEC analysis.

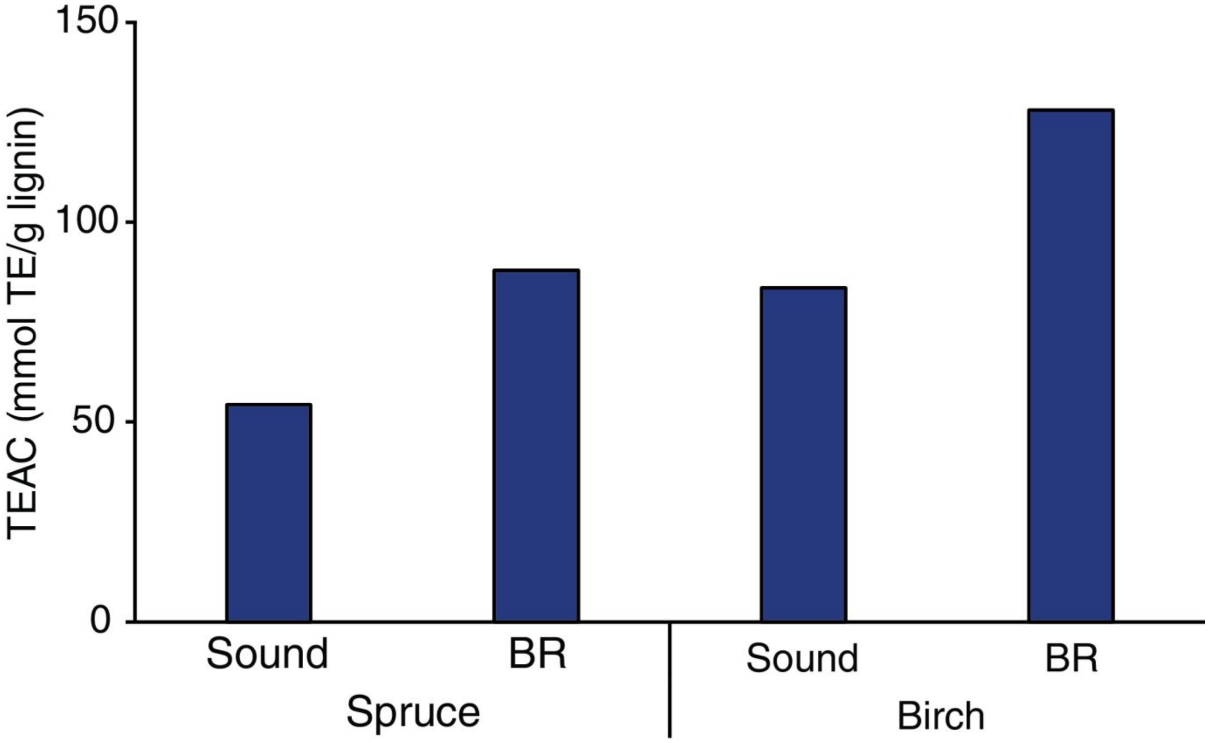

Figure S8. Antioxidant capacity of lignin isolates. The Trolox Equivalent Antioxidant Capacity (TEAC) method was used to determine the antioxidant capacity of lignin isolated from sound and brown-rot decayed spruce and birch. Data are single measurements. Method validation indicated an error of < ±1%, and therefore the observed differences between sound and brown-rot decayed wood are assumed to be significant.

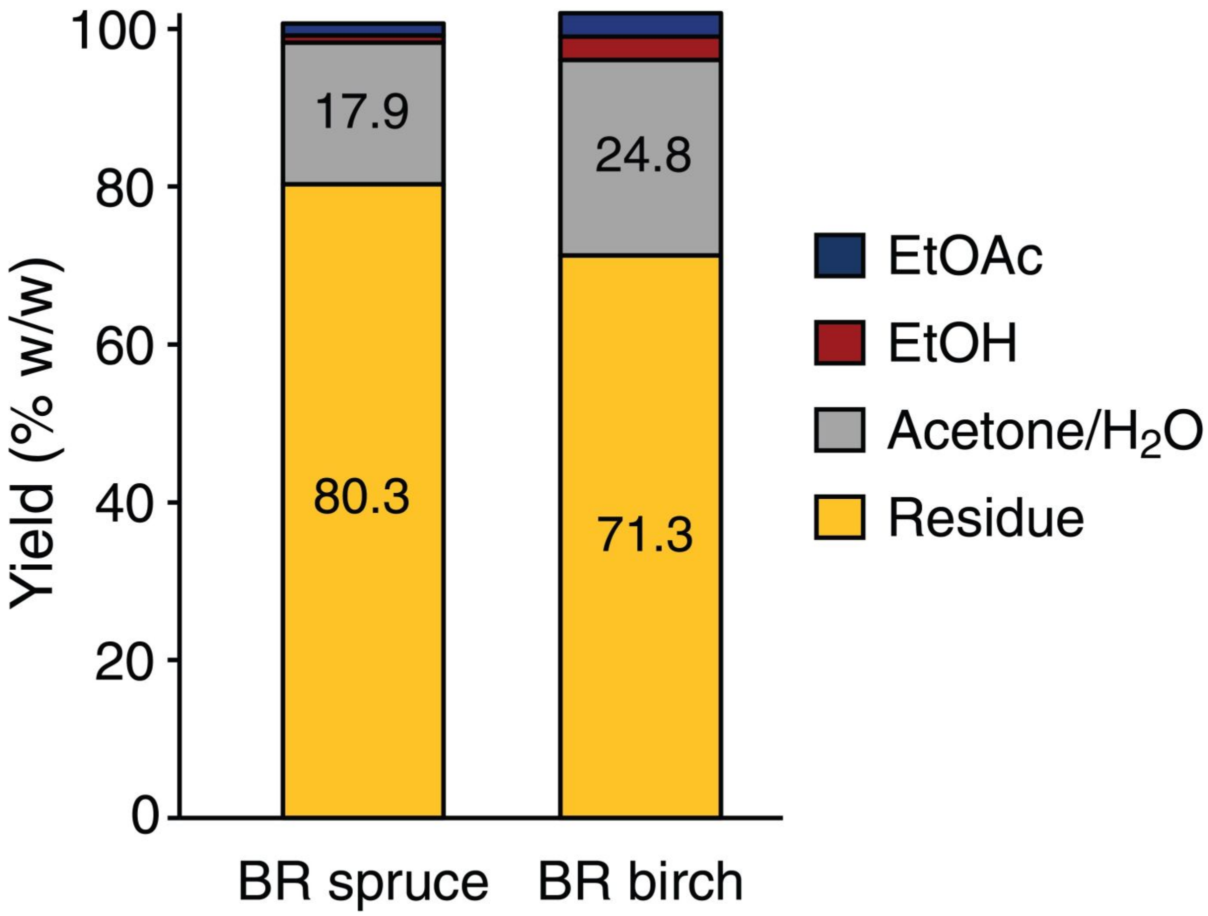

Figure S9. Sequential solvent fractionation of brown-rot decayed spruce and birch. Relative mass (dry weight) of fractions after sequential solvent fractionation of brown-rot decayed spruce and birch with ethyl acetate (EtOAc), ethanol (EtOH) and 80% (v/v) acetone in water (Ace/H_2O).

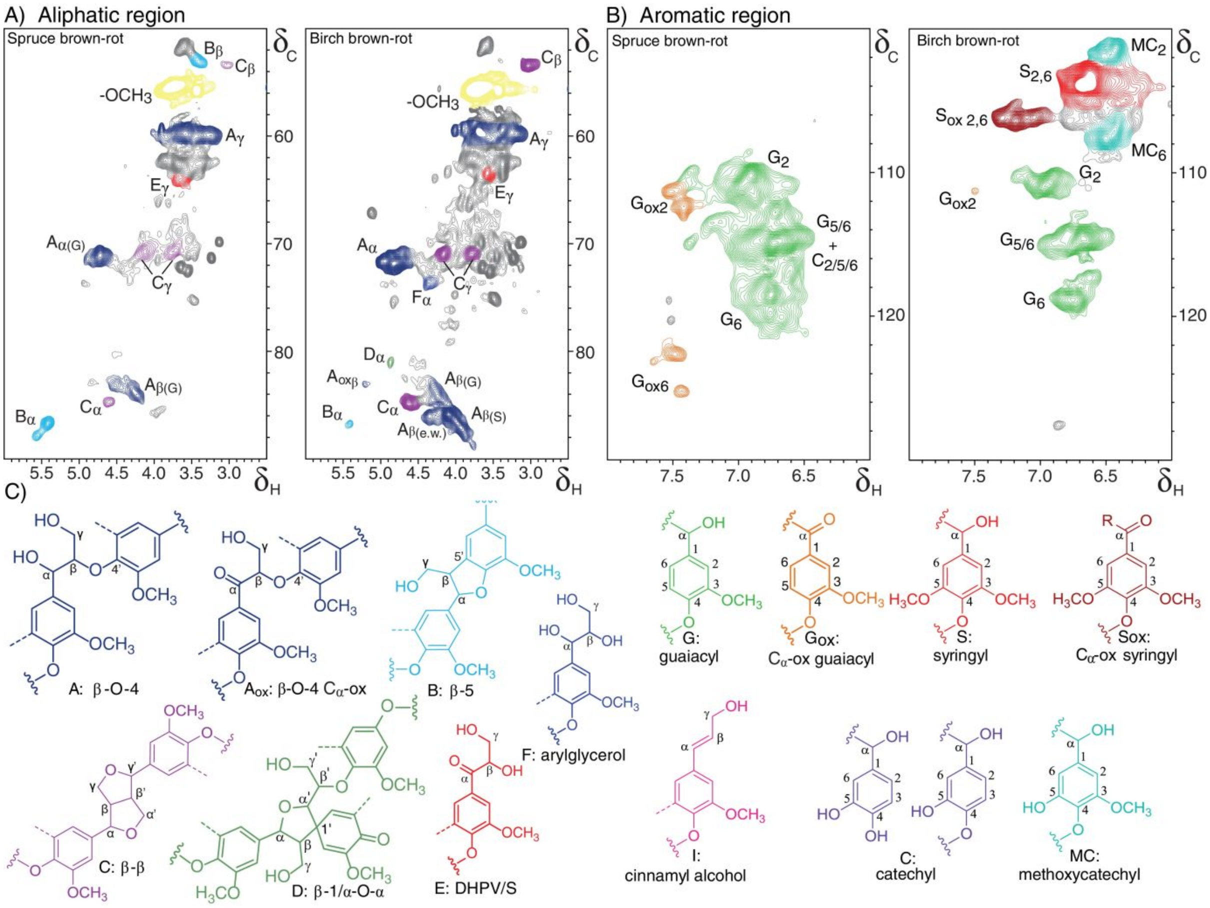

Figure S10. HSQC NMR spectra of acetone/H_2O extracts of brown-rot decayed wood. The spectra show the aliphatic (A) and aromatic (B) regions. Annotated substructures (C) are shown with subscripted numbers and Greek letters indicating which carbon in the annotated substructure the signal originates from. Annotated substructures have colors corresponding to colored signals in A and B. Dashed lines indicate -H (guaiacyl) or -OCH3 (syringyl), while the main position for further coupling is indicated with wavy lines. Unassigned peaks are shown in grey.

Table S1. Identity and structural classification of lignin-derived pyrolysis products detected using ^{13}C-IS pyrolysis-GC-MS.

#	Compound	CAS	Retention time (min)	Structural feature	Sidechain length	M_w ^{12}C ($g\cdot mol^{-1}$)	Quan ^{12}C [M-*e*]	ionM_w ^{13}C ($g\cdot mol^{-1}$)	Quan ion ^{13}C [M-*e*]
1	phenol	108952	9.61	H, unsub.	0	94	94.04132	100	100.06145
2	guaiacol	90051	9.91	G, unsub.	0	124	124.05188	131	115.04853
3	2-methylphenol	95487	10.87	H, methyl	C_α	108	108.05698	115	115.08045
4	4-methylphenol (+3-MP)	106445	11.83	H, methyl	C_α	108	107.04914	115	114.07263
5	4-methylguaiacol	93516	12.58	G, methyl	C_α	138	138.06753	146	146.09437
6	2,4-dimethylphenol	105679	13.06	H, methyl	C_α	122	107.04914	130	114.07263
7	4-ethylphenol	123079	14.12	H, misc.	C_β	122	107.04914	130	114.07263
8	4-ethylguaiacol	2785899	14.73	G, misc.	C_β	152	137.05971	161	145.08654
9	4-vinylguaiacol	7786610	16.20	G, vinyl	C_β	150	150.06753	159	159.09754
10	4-vinylphenol	2628173	16.36	H, vinyl	C_β	120	120.05697	128	128.08381
11	eugenol	97530	16.80	G, misc.	C_γ	164	164.08318	174	174.11673
12	4-propylguaiacol	2785877	16.90	G, misc.	C_γ	166	137.05971	175	145.08654
13	syringol	91101	17.52	S, unsub.	0	154	154.06245	162	162.08928
14	*cis*-isoeugenol	97541	18.18	G, misc.	C_γ	164	164.08318	174	174.11673
15	4-propenylphenol	539128	19.14	H, misc.	C_γ	134	133.06479	143	142.09498
16	*trans*-isoeugenol	97541	19.49	G, misc.	C_γ	164	164.08318	174	174.11673
17	4-methylsyringol	6638057	19.74	S, methyl	C_α	168	168.07810	177	177.10829
18	vanillin	121335	19.83	G, C_α-O	C_α	152	151.03897	160	159.06581
19	4-propyneguaiacol	-	20.23	G, misc.	C_γ	162	162.06753	172	172.10108
20	4-alleneguaiacol	-	20.47	G, misc.	C_γ	162	162.06753	172	172.10108
21	homovanillin	5603242	21.25	G, C_β-O	C_β	166	137.05971	175	145.08654
22	4-ethylsyringol	14059928	21.46	S, misc.	C_β	182	167.07022	192	176.10046
23	vanillic acid methyl ester	3943746	21.72	G, C_α-O	C_α	182	182.05736	191	191.08766
24	acetovanillone	498022	21.73	G, C_α-O	C_β	166	151.03897	175	159.06581
25	4-hydroxybenzaldehyde	123080	22.52	H, C_α-O	C_α	122	121.02848	129	128.05189
26	4-vinylsyringol	28343228	22.80	S, vinyl	C_β	180	180.07810	190	190.11164
27	guaiacylacetone	2503460	22.92	G, C_β-O	C_γ	180	137.05971	190	145.08654
28	4-allylsyringol	6627889	23.22	S, misc.	C_γ	194	194.09373	205	205.13065
29	propiovanillone	1835149	23.67	S, C_α-O	C_γ	180	151.03897	190	159.06581
30	guaiacyl vinyl ketone	-	23.94	G, C_α-O	C_γ	178	151.03897	188	159.06581
31	guaiacyl diketone	2034608	24.14	G, C_α-O, C_β-O	C_γ	194	151.03897	204	159.06581
32	cis-4-propenylsyringol	26624135	24.36	S, misc.	C_γ	194	194.09373	205	205.13065
33	4-propynesyringol	-	25.03	S, misc.	C_γ	192	192.07810	203	203.11500
34	4-allenesyringol	-	25.24	S, misc.	C_γ	192	192.07810	203	203.11500
35	trans-4-propenylsyringol	26624135	25.71	S, misc.	C_γ	194	194.09373	205	205.13065
36	dihydroconiferyl alcohol	2305137	25.72	S, C_γ-O	C_γ	182	137.05971	192	145.08654
37	syringaldehyde	134963	26.15	S, C_α-O	C_α	182	182.05736	191	191.08755
38	cis-coniferyl alcohol	458355	26.30	G, C_γ-O	C_γ	180	137.05971	190	145.08654
39	cis-coumaryl alcohol	3690059	26.50	H, C_γ-O	C_γ	150	107.04914	159	114.07263
40	homosyringaldehyde	-	27.13	S, C_β-O	C_β	196	167.07027	206	176.10046
41	acetosyringone	2478388	27.57	S, C_α-O	C_β	196	181.04954	206	190.07973
42	syringic acid methyl ester	884355	27.58	S, C_α-O	C_α	212	212.06793	222	222.10147
43	trans-coumaryl alcohol	3690059	27.58	H, C_γ-O	C_γ	150	107.04914	159	114.07263
44	trans-coniferyl alcohol	458355	28.04	G, C_γ-O	C_γ	180	137.05971	190	145.08654
45	trans-coniferaldehyde	458366	28.37	G, C_γ-O	C_γ	178	147.04406	188	156.07425
46	syringylacetone	19037582	28.47	S, C_β-O	C_γ	210	167.07027	221	176.10046
47	propiosyringone	5650431	29.16	S, C_α-O	C_γ	210	181.04954	221	190.07973
48	syringyl diketone	6925651	29.25	S, C_α-O, C_β-O	C_γ	224	181.04954	235	190.07973
49	syringyl vinyl ketone	-	29.43	S, C_α-O	C_γ	208	181.04954	219	190.07973
50	dihydrosinapyl alcohol	20736258	31.01	G, C_γ-O	C_γ	212	168.07841	223	177.10829
51	cis-sinapyl alcohol	537337	31.48	S, C_γ-O	C_γ	210	167.07027	221	176.10046
52	trans-sinapyl alcohol	537337	33.23	S, C_γ-O	C_γ	210	167.07027	221	176.10046
53	trans-sinapaldehyde	4206580	33.40	S, C_γ-O	C_γ	208	208.07301	219	219.10994

Table S2. Annotation of catechol and methoxycatechol pyrolysis products detected using ^{13}C-IS pyrolysis-GC-MS.

Retention time (min)	Quan ion ^{12}C [*m/z*]	Annotation
16.04	140.04735	methoxycatechol
16.62	124.05243	4-methylcatechol
17.45	110.03623	catechol
18.12	140.04735	methoxycatechol derivative
18.40	124.05243	catechol derivative
19.38	124.05243	catechol derivative
21.08	140.04735	methoxycatechol derivative
21.67	140.04735	methoxycatechol derivative

Table S3. Composition of wood samples. The table shows the content of carbohydrates, lignin, and protein in sound and brown-rot decayed (BR) wood as percentage of the total dry mass, as well as the total accounted fraction of the dry matter. AIL, acid insoluble lignin; ASL, acid soluble lignin. Numbers are shown as average ± standard deviation (n=2 for protein, n=3 for others).

Wood	Carbohydrates	Protein	AIL[a]	ASL	Lignin (py-GC-MS)	Fraction accounted[b]
**Spruce**	64±2	n.d.	27.5±0.9	0.8±0.1	31 ± 2	92 ± 2
**BR Spruce**	5±1	1.91±0.08	80.8±0.7	2.2±0.1	64 ± 4	90 ± 1
**Birch**	64±4	n.d.	22.1±0.1	4.7±0.5	30 ± 2	91 ± 4
**BR Birch**	19±1	2.88±0.04	65.1±0.3	5.3±0.2	61 ± 1	91 ± 1

[a]Corrected for protein content in the AIL fraction (0.8% in BR spruce and 1.6% in BR birch)
n.d.: not detected/below limit of detection.

[b] sum of carbohydrates, protein, AIL, ASL

Table S4. Constituent anhydro-monosaccharide composition. Calculated content of monosaccharides in sound and brown-rot decayed wood as percentage of the total dry matter. Numbers are shown as average ± standard deviation (n=3).

Wood	Arabinose	Galactose	Glucose	Xylose	Mannose
**Spruce**	1.04±0.07	1.48±0.06	43 ±2	6.1±0.3	12.9±0.3
**BR Spruce**	0.18±0.08	0.29±0.09	2.0±0.5	1.9±0.3	0.96±0.5
**Birch**	0.51±0.03	0.80±0.01	39 ±2	23 ±2	1.79±0.05
**BR Birch**	0.11±0.04	0.22±0.04	8.2±0.7	7.2±0.3	3.0±0.6

Table S5. Hydroxyl content (mmol/g) of lignin isolated from brown-rot decayed wood as determined by ^{31}P NMR. Aliphatic: Aliphatic OH groups, Benzoic acid: COOH groups in aromatic rings. S+G cond.: Condensed syringyl (S) + guaiacyl (G) units. G: Non-condensed guaiacyl units. H+MC+C: hydroxyl groups from p-hydroxyphenyl (H), methoxy-catechols (MC), catechols (C). Total phenolic: Sum of all phenolic hydroxyl groups (condensed + non-condensed)

		**Aliphatic**	**Benzoic acid**	**S+G cond.**	**G**	**H+MC+C**	**Total phenolic**
**Spruce**	Sound	2.98	0.17	0.47	0.42	0.08	0.96
	Brown-rot	3.28	0.33	0.89	1.14	0.20	2.23
**Birch**	Sound	4.32	0.18	0.65	0.31	0.09	1.05
	Brown-rot	3.47	0.28	0.93	0.65	0.15	1.83

Table S6. Molecular weight distribution of lignin isolated from brown-rot decayed spruce and birch, and acetone/H_2O extracts of these, as determined by alkaline SEC. Lignin isolated from sound wood could not be analyzed by SEC due to incomplete solubility. M_w: weight-average molecular weight. M_n: number-average molecular weight. Đ: dispersity/polydispersity index, which is the ratio of M_w to M_n.

		**M_w (g/mol)**	**M_n (g/mol)**	**Đ (M_w/M_n)**
**Spruce**	Brown-rot	6450	1690	3.8
	Acetone/H_2O	2100	860	2.4
**Birch**	Brown-rot	4990	1370	3.6
	Acetone/H_2O	2010	820	2.5

Table S7. Structural characterization of acetone/H_2O extracts of brown-rot decayed spruce and birch by semi-quantitative HSQC NMR (400 MHz). G_{ox}/S_{ox}: guaiacyl/syringyl units oxidized on the Cα-carbon. G_{cond}: guaiacyl units involved in condensed linkages. MC: methoxycatechyl units. MC_{ox}: methoxycatechyl units oxidized on the Cα-carbon, "ar" refers to aromatic rings.

**Subunits (%)**	**BR spruce acetone/H_2O**	**BR birch acetone/H_2O**
G	69.4	17.8
G_{cond}	17.0	0.0
G_{ox}	13.5	0.0
S	0.0	59.4
S_{ox}	0.0	10.1
MC	0.0	12.7
MC_{ox}	0.0	0.0
S/G	-	3.9
Interunit linkages (per 100 ar)		
β-O-4 aryl ether	25.5	49.6
β-5 phenylcoumaran	10.9	0.6
β-β resinol	1.5	5.8
End units (per 100 ar)		
Cinnamaldehyde	1.4	2.3
Benzaldehyde	5.9	1.7
Ring substituents (per 100 ar)		
Methoxyl[a]	90.8 (88.5)[b]	136.4

[a]corrected for the overlapping residual EtOH solvent peak; [b]the value in parentheses is based on the total aromatic region to account for C2, C5, C6, G5, G6 overlap. "ar" refers to the aromatic rings.
